# Negative binomial mixed models for analyzing longitudinal CD4 count data

**DOI:** 10.1038/s41598-020-73883-7

**Published:** 2020-10-07

**Authors:** Ashenafi A. Yirga, Sileshi F. Melesse, Henry G. Mwambi, Dawit G. Ayele

**Affiliations:** 1 School of Mathematics, Statistics, and Computer Science, University of KwaZulu-Natal, Private Bag X01, Scottsville, Pietermaritzburg, 3209 South Africa; 2 Institute of Human Virology, School of Medicine, University of Maryland, Baltimore, MD USA

**Keywords:** Biomarkers, Infectious diseases, Epidemiology

## Abstract

It is of great interest for a biomedical analyst or an investigator to correctly model the CD4 cell count or disease biomarkers of a patient in the presence of covariates or factors determining the disease progression over time. The Poisson mixed-effects models (PMM) can be an appropriate choice for repeated count data. However, this model is not realistic because of the restriction that the mean and variance are equal. Therefore, the PMM is replaced by the negative binomial mixed-effects model (NBMM). The later model effectively manages the over-dispersion of the longitudinal data. We evaluate and compare the proposed models and their application to the number of CD4 cells of HIV-Infected patients recruited in the CAPRISA 002 Acute Infection Study. The results display that the NBMM has appropriate properties and outperforms the PMM in terms of handling over-dispersion of the data. Multiple imputation techniques are also used to handle missing values in the dataset to get valid inferences for parameter estimates. In addition, the results imply that the effect of baseline BMI, HAART initiation, baseline viral load, and the number of sexual partners were significantly associated with the patient’s CD4 count in both fitted models. Comparison, discussion, and conclusion of the results of the fitted models complete the study.

## Introduction

After it is identified by scientists as the human immunodeficiency virus (HIV) and the cause of acquired immunodeficiency syndrome (AIDS) in 1983, HIV has spread persistently, triggering one of the most severe pandemics ever documented in human history. More than 75 million individuals have been infected with HIV, more than 32 million individuals have perished due to AIDS-related causes since the pandemic started, and 7000 new infections are reported daily. Worldwide, 37.9 million [32.7–44.0 million] individuals were HIV positive at the end of 2018. Approximately 0.8% [0.6–0.9%] of grownup persons in the age range fifteen to forty-nine years worldwide are living with HIV, even though the problem of the epidemic continues to vary sizably between nations and regions^1^. Despite recent progressions in HIV prevention, care, and treatment, which has modestly decreased the total number of new infections and deaths every year, AIDS and AIDS-related illnesses are still among the driving causes of loss of life globally. Sub-Saharan Africa and Southern Africa, in specific, is right now the region most influenced by HIV/AIDS in the world^2^. The HIV crisis in South Africa is critical. Since South Africa is at the epicenter of the HIV/AIDS epidemic, South African concerns are worldwide concerns, and lessons learned in South Africa are lessons for the universal community.

HIV/AIDS and other STD have an obliterating effect on women’s health, especially the well-being of younger ladies. “The consequences of HIV/AIDS attain beyond women’s health to their part as mothers and caregivers and their commitment to the economic support of their families. The social, development, and health consequences of HIV/AIDS and other sexually transmitted illnesses should be seen from a gender perspective”^3–5^. “It needs to be emphasized that, except for sex-specific issues, treatment algorithms for HIV-Infected women do not differ from men’s. Dialogs about the changing epidemiology of HIV will provide the clinician a system to decide who may be at high risk and to clarify the application of rules to avoid sequential HIV transmission. Even though antiretroviral recommendations presently remain the same for men and women, the survey of discoveries for early HIV infection and the individual difference in CD4 cell count/viral load of HIV-infected patient will permit the clinician to interpret prospective information appropriately and to address deception or distortion of this information by patients”^6–8^.

“CD4 cell counts deliver a sign of the wellbeing of an individual immune system (body’s natural defense system against pathogens, infections, and illnesses). It also provides information about disease progression. CD4 cells are white blood cells (in a cubic millimeter of blood) that play an essential role in the immune system. A higher number shows a stronger immune system. The CD4 cell counts of a person who does not have HIV can be anything between 500 and 1500. Individuals living with HIV who have a CD4 count over 500 are usually in good health. Individuals living with HIV who have a CD4 cell count below 200 are at high risk of developing serious illnesses^9^. HIV treatment is prescribed for all individuals living with HIV. It is particularly critical for patients with low CD4 count, which is superior to start treatment sooner, rather than later”^6^. The study of HIV infection at the acute stage is essential to the plan and advancement of HIV antibodies and techniques to attain an undetectable level of the infection without ART or a functional remedy. Researchers have managed to find out about the early events following infection by diagnosing HIV within a month, weeks, or even days of infection. Moreover, humans dwelling with HIV who are not on treatment or who are not virally suppressed can also have a compromised immune system (measured by a low CD4 count) that makes them at risk of the new and ongoing coronavirus disease 2019 (COVID-19) pandemic, opportunistic infections, and underlying illnesses. Whereas analysts accept that early diagnosis and prompt treatment of HIV are the stepping stones to a functional remedy, more studies are required to understand better the adaptive, innate, and host responses that regulate viral load set-point and subsequently diagnosis and infectiousness.

Count data are ubiquitous in public health investigations. This sort of data assumes only positive integer values (i.e., 0, 1, 2, …). The most commonly used method for count data is the Poisson distribution and its related enhancement, such as the Poisson-gamma mixture, which considers over-dispersion and heterogeneity in the model. This paper’s main contribution is the inclusion of the links between CD4 cell count and influencing covariates of biometric and demographic factors. Therefore, this study aims to cope with the statistical challenges of over-dispersion and incorporate within-subject correlation structures by applying NBMMs to longitudinal CD4 count data from the CAPRISA 002 AI Study and also detecting factors that are significantly associated with the response variable.

## Materials and methods

### Data description

This study makes use of data from the CAPRISA 002 AI Study. The study was conducted on HIV-infected women at the Doris Duke Medical Research Institute (DDMRI) at the Nelson R Mandela School of Medicine of the University of KwaZulu-Natal in Durban, South Africa. Between August 2004 and May 2005, CAPRISA introduced a cohort study recurring high-risk HIV negative women to a follow-up study. In the case of the data used in this paper as part of an ongoing study, women infected with HIV are enrolled in the study early, followed intensely, and monitored carefully to examine disease progression and CD4 count/viral load evolution. One can refer to studies by Van Loggerenberg et al.^10^ and Mlisana et al.^11^ for details on the design, development, and procedures of the study population.

### Methods

A linear model consists of a response variable ${\text{Y}}$, which is assumed to be normally distributed, and several predictors ($x_{1} , x_{2} , \ldots ,x_{p}$). Multiple regression analysis studies the linear relationships among two or multiple independent variables and one dependent (response) variable. The multiple regression model is given by

$$y_{i} = \beta_{0} + \beta_{1} x_{i1} + \beta_{2} x_{i2} + \ldots + \beta_{p} x_{ip} + \varepsilon_{i} = \beta_{0} + {\varvec{x}}_{{\varvec{i}}}^{\user2{^{\prime}}} {\varvec{\beta}} + \varepsilon_{i} = \beta_{0} + \user2{\beta^{\prime}x}_{{\varvec{i}}} + \varepsilon_{i} , i = 1, \ldots , n.$$

where $y_{i}$ is the response variable, ${\varvec{x}}_{{\varvec{i}}}$ is a $p \times 1$ vector of explanatory variables, $\beta_{0}$ is the intercept, ${\varvec{\beta}}$ is a $p \times 1$ vector of unknown regression coefficients, and $\varepsilon_{i} \mathop \sim \limits^{iid} N\left( {0, \sigma^{2} } \right)$, which is a random error of observation $i.$ We can extend these multiple linear regression model ideas to generalized linear models (GLM) where the distribution of the outcome variable can include distributions other than normal. The outcome $y_{i}$ can be continuous, dichotomous, count, ordinal, categorical, and so on as long as its distribution is from the exponential family. The exponential family of distributions incorporates numerous distributions that are valuable for viable modeling such as Poisson and Negative Binomial for count data; Binomial, Bernoulli, and Geometric for discrete data; Gamma, Normal, Inverse Gaussian, Beta, and Exponential for the study of continuous response data set. More details on exponential family and related topics can be found in Dobson et al.^12^.

A Poisson process is mainly used as an initial point for modeling the stochastic difference of count data around a theoretical expectation. However, in reality, the patient’s data have more differences than using the Poisson distribution. The model’s over-dispersion is accounted for because of different model assumptions about the variance changes with the expectation. To the value of statistical inferences, the choice of these assumptions has major consequences. Therefore, the negative binomial distribution parameterization is proposed because the method introduces various quadratic mean–variance relationships, incorporating the ones assumed in the most commonly used approaches.

The Poisson regression is a commonly-used statistical model for $n$ responses $y_{1} , \ldots ,y_{n}$ whose domain is non-negative integer values. Each $y_{i}$ is modeled as an independent Poisson ($\lambda_{i}$) random variable and distributed as $y_{i} \mathop \sim \limits^{iid}$ Poisson ($\lambda_{i}$), where the parameter $\lambda_{i}$ controls the count rate in the $i{\text{th}}$ outcome. Thus, a model for the Poisson rate parameter $\lambda_{i}$ is given by

$$\ln \lambda_{i} = \beta_{0} + \beta_{1} x_{i1} + \ldots + \beta_{p} x_{ip} = \beta_{0} + \mathop \sum \limits_{j = 1}^{p} \beta_{j} x_{ij}$$

or equivalently,

$$\lambda_{i} = e^{{\beta_{0} + \beta_{1} x_{i1} + \ldots + \beta_{p} x_{ip} }} = e^{{\beta_{0} + \mathop \sum \limits_{j = 1}^{p} \beta_{j} x_{ij} }}$$

where $x_{i1} , \ldots ,x_{ip}$ are a set of $p$ explanatory variables, and $\beta = \left( {\beta_{0} , \ldots ,\beta_{p} } \right)$ are the regression coefficients. The probability mass function (pmf) of the Poisson random variable with parameter $\lambda_{i}$ is given by

1 $$f\left( {y_{i} , {\lambda }_{i} } \right) = \frac{{e^{{ - {\lambda }_{i} }} {\lambda }_{i}^{{y_{i} }} }}{{y_{i} !}}, y_{i} = 0, 1, 2, \cdots$$

Since $y_{i} \mathop \sim \limits^{iid}$ Poisson ($\lambda_{i}$), as a consequence, the likelihood function is equal to the product of their pmf and the log-likelihood function can be derived by taking the natural logarithm of the likelihood function, become

$$= \mathop \sum \limits_{i = 1}^{n} \left[ {y_{i} \ln \left( {\lambda_{i} } \right) - \lambda_{i} - \ln y_{i} !} \right]$$

where $\lambda_{i}$ is defined in terms of $\beta_{0} , \ldots ,\beta_{p}$ and the covariates $x_{i1} , \ldots ,x_{ip}$ in Eq. (1), the log-likelihood function can be expressed as

$$\begin{aligned} \ell \left( {\beta_{0} , \ldots ,\beta_{p} } \right) & = \mathop \sum \limits_{i = 1}^{n} \left[ {y_{i} \left( {\mathop \sum \limits_{j = 0}^{p} \beta_{j} x_{ij} } \right) - e^{{\mathop \sum \limits_{j = 0}^{p} \beta_{j} x_{ij} }} - \ln y_{i} !} \right] \\ & = \mathop \sum \limits_{i = 1}^{n} \left\{ {y_{i} {\varvec{x}}_{{\varvec{i}}}^{\user2{^{\prime}}} {\varvec{\beta}} - {\exp}\left( {{\varvec{x}}_{{\varvec{i}}}^{\user2{^{\prime}}} {\varvec{\beta}}} \right) - \ln y_{i} !} \right\}. \\ \end{aligned}$$

For a presentation of efficient computational methods for maximizing $\hat{\user2{\beta }}$, and $V\left[ {\hat{\user2{\beta }}} \right]$, see Hilbe^13^.

Suppose the response variable $y_{i}$ follows a Poisson distribution with mean $\lambda_{i}$ and there is no over- or under-dispersion, then $var\left( {y_{i} } \right) = \lambda_{i}$ that is the mean and variance are equal. The restriction (mean = variance) may not be satisfied with many real-world data. Sometimes the variance is greater than the mean, and this phenomenon is called over-dispersion. One such model that works in such a condition is the negative binomial regression model.

If there is over-dispersion $var\left( {y_{i} } \right) = \Phi \lambda_{i}$ and $\Phi > 1$. While if there is under-dispersion $var\left( {y_{i} } \right) = \Phi \lambda_{i}$ and $\Phi < 1$ that is $var\left( {y_{i} } \right) > E\left( {y_{i} } \right)$, in this case, the Poisson distribution is no longer suitable. The method of moments solution for the dispersion parameter $\Phi$ is found from the sample relation that is $var\left( {y_{i} } \right) = \hat{\Phi }\overline{y}$. Therefore, $\hat{\Phi } = \frac{{{\text{var}} \left( {y_{i} } \right)}}{{\overline{y}}}$, and then if $\hat{\Phi } > 1$, evidence of over-dispersion. Data may be over-dispersed if the Pearson Chi-Square (${\chi }^{2}$)/DF value is greater than 1.0. In general, when the value is greater than 2.0, it is an indication of over-dispersion, it requires remedial action^13,14^. Over-dispersed data can lead to underestimated SEs and inflated test statistics^13–16^. In such circumstances, the negative binomial model can be utilized, and therefore the formulation can be expressed as $y_{i} \sim NB\left( {\mu_{i} , \mu_{i} \left[ {1 + \alpha \mu_{i} } \right]} \right)$, where $\alpha (\alpha > 0)$ can be utilized to add flexibility, and plays the role of the scale parameter, for variance independently of the mean. The negative binomial model is a generalization of the Poisson model, which relaxes the restrictive assumption that the variance and mean are equal^13–15^. Just like the Poisson model, the negative binomial model is commonly utilized as a distribution for count data; however, it allows a variance higher than its mean. The most contrast between the NB and Poisson models is the extra parameter (scale parameter) that controls for the over-dispersion and, thus, the determination of the likelihood functions related to them^13,14^. Estimation of the parameters can be accomplished through likelihood maximization by employing a nonlinear optimization method^13,14^. The parametrization process of the negative binomial model is discussed later.

In general, for the inference of count data, the four most commonly used statistical model distributions are the Poisson, Negative Binomial, Hurdle, and Zero-Inflated regression models. The NB model addresses the issue of over-dispersion by including a dispersion parameter that relaxes the presumption of equal mean and variance in the distribution whilst the Hurdle and Zero-Inflated regression models are utilized to handle the distribution of count outcome with excess zeroes^17–21^.

The generalized linear model fails to consider the dependence of repeated observations over time. That means when data are measured repeatedly like CD4 counts of several individuals over time, the assumption of independence is no longer reasonable. Therefore, it is necessary to extend the GLM to generalized linear mixed-effects models, including a subject-specific random effect introduced in the *linear predictor* to seize the dependence.

Recall the linear mixed model:

$$y_{ij} = \left( {\beta_{0} + b_{i0} } \right) + \left( {\beta_{1} + b_{i1} } \right)X_{1ij} + \ldots + \left( {\beta_{p} + b_{ip} } \right)X_{pij} + \varepsilon_{ij},$$

where $y_{ij}$ is an outcome variable, P is the predictor variable, $\beta_{1} , \ldots , \beta_{p}$ are fixed effects, $b_{i1} , \ldots , b_{ip}$ are random effects and $\varepsilon_{ij}$’s are residuals.

Suppose we want to generalize the above model. In that case, we do not need to assume that the outcome variable is normally distributed even after a transformation, such as the square root transformation for the CD4 count. However, it has to follow a distribution from the exponential family; at that point, we can combine the mixed model’s idea with the generalized linear model. For instance, if $y_{ij}$ is a count, we could look at Poisson regression. Hence the Poisson linear mixed model gets to be

$$\log \left( {E\left( {y_{ij} } \right)} \right) = \beta_{0} + \beta_{1} x_{1ij} + \cdots + \beta_{p} x_{pij} + b_{0} + b_{1} x_{1ij} + \cdots + b_{p} x_{pij}$$

In matrix notation form, the conditional mean of $y_{ij}$ rely on fixed and random effects via the subsequent linear predictor:

$$\log \left\{ {E(y_{ij} |{\varvec{b}}_{{\varvec{i}}} )} \right\} = \eta_{ij} = {\varvec{x}}_{ij}^{^{\prime}} {\varvec{\beta}} + {\varvec{z}}_{ij}^{^{\prime}} {\varvec{b}}_{i} .$$

where $y_{ij}$’s are independent and have a Poisson distribution, conditional on a vector of random effects $b_{i}$, with $var\left( {y_{ij} {|}{\varvec{b}}_{{\varvec{i}}} } \right) = E\left( {y_{ij} {|}{\varvec{b}}_{{\varvec{i}}} } \right), \left( {i.e., \Phi = 1} \right)$, and ${\varvec{x}}_{ij}^{^{\prime}} = {\varvec{z}}_{ij}^{^{\prime}} = \left( {1, t_{ij} } \right)$. That is, the conditional mean of $y_{ij}$ is associated with the linear predictor via a log link function, which is an example of a log-linear mixed-effects model^22,23^.

Several methods are available to estimate the parameters ($\beta_{i}$’s and $b_{i}$’s) in GLMMs, which includes marginal quasi-likelihood (MQL), penalized (predictive) quasi-likelihood (PQL), the Laplace approximation, the Gauss-Hermite quadrative and the Markov Chain Monte Carlo (MCMC) method^24–27^. Our preference is for the Laplace approximation due to the fewer limitations than the Adaptive quadrature (method = quad). It is accurate, fast, and gives us the plausibility to use the likelihood and information criteria^26,28,29^. However, R-side random effects are not supported for method = laplace or method = quad in the Proc Glimmix statement. Instead, Proc Glimmix uses a random statement and the *residual option* to model repeated (R-side) effects.

“The parameter estimates based on the mixed-effects negative binomial model are not exceptionally different from those based on mixed-effects Poisson model. However, the Poisson model underestimates the SEs when over-dispersion is present, leading to improper inference. A straightforward way to select between these two models is to compare them based on a few criteria, such as AIC and BIC”^23^. Where for the ICs, a lower value means that the model fits better than the competing model. We may, moreover, compare models utilizing $- 2loglikelihood$, and the *likelihood ratio test* for nested models. To some degree, parameters in GLMMs have different interpretations than parameters in the conventional marginal models. In GLMMs, the regression coefficients have subject-specific interpretations. Especially, they characterize the impact of variables on a particular subject’s mean response. More specifically, the $\beta ^{\prime}s$ are interpreted in terms of the effects of within-subject changes in explanatory variables on changes in an individual’s transformed mean response, while holding the remaining covariates constant. Accordingly, $\beta_{j}$ is interpreted as the change in an individual’s log of response for a unit increase in ${\varvec{x}}_{{{\varvec{ij}}}}$, while holding other fixed variables constant for that individual. Since the elements of the fixed effects, $\beta_{j}$, have interpretations conditional on $b_{i}$, the $i{\text{th}}$ individual’s random effects, they are regularly known as subject-specific regression coefficients. “Thus, GLMMs are most useful when the main scientific objective is to make inferences about individuals instead of the population average effects; the population averages are the targets of inference in marginal models”^22^.

The negative binomial (NB) distribution, also the result of a Poisson–Gamma mixture, has vast applications as a model for count data, especially for data showing over-dispersion. It has properties that are comparable to the Poisson model, as discussed above, in which the outcome variable $Y_{i}$ is modeled as a Poisson variable with a mean ${\lambda }_{i}$ where the model error is assumed to follow a Gamma distribution. The Poisson-Gamma mixture model was developed to account for over-dispersion that is widely observed in discrete or count data^30^. The pdf of the NB distribution is frequently expressed in terms of the mean $\lambda$ and dispersion parameter $\theta$ such that the probability of observing a non-negative integer $k$, which was given by Demidenko^31^ parameterization of the negative binomial regression, discussed as follows:

If $Y$ takes discrete values with the conditional Poisson distribution: $P_{r} \left( {Y = k|{\lambda }} \right) = \frac{{e^{{ - {\lambda }}} {\lambda }^{k} }}{k!}$, where ${\lambda } > 0$, ${\lambda }\sim {{Gamma}}\left( {{\alpha },{{ \theta }}} \right)$ then the pdf of a two-parameter, ${\alpha },\,\, and\,\, {{ \theta }}$, Gamma distribution is given by:

2 $$f\left( {{\lambda };{\alpha },{{ \theta }}} \right) = \frac{{{\lambda }^{\alpha - 1} e^{{ - {\lambda }/{\theta }}} }}{{\theta^{\alpha } \Gamma \left( \alpha \right)}},\quad {\lambda } > 0,\quad \alpha > 0,\quad {\theta } > 0$$

Thus, the negative binomial (Poisson–Gamma) model can be defined as:

3 $$f\left( {Y|{\lambda }} \right) = \frac{{e^{{ - {\lambda }}} {\lambda }^{k} }}{k!}\frac{{{\lambda }^{\alpha - 1} e^{{ - {\lambda }/{\theta }}} }}{{\theta^{\alpha } \Gamma \left( \alpha \right)}}$$

It has also been defined in the literature as:

4 $$= \left( {\begin{array}{*{20}c} {\alpha + k - 1} \\ k \\ \end{array} } \right)\left( {\frac{\theta }{1 + \theta }} \right)^{k} \left( {\frac{1}{1 + \theta }} \right)^{\alpha } = \frac{{ \Gamma \left( {\alpha + k} \right)}}{k! \Gamma \left( \alpha \right)}\left( {\frac{\theta }{1 + \theta }} \right)^{k} \left( {\frac{1}{1 + \theta }} \right)^{\alpha } ,$$

where the binomial coefficient is computed as $$\left( {\begin{array}{*{20}c} {\alpha + k - 1} \\ k \\ \end{array} } \right) = \frac{{\left( {\alpha + k - 1} \right)\left( {\alpha + k - 2} \right) \ldots \alpha }}{k!} = \frac{{\left( {\alpha + k - 1} \right)!}}{{k!\left( {\alpha - 1} \right)!}}$$. Note that for a positive integer $\alpha$, we have $\Gamma \left( \alpha \right) = \left( {\alpha - 1} \right)!$.

For negative binomial distribution, $E\left( y \right) = \alpha \theta$, and $var\left( y \right) = \alpha \theta \left( {1 + \theta } \right)$. For Poisson distribution, the mean and variance are equal, but the variance is higher than the mean by $\alpha \theta^{2}$ for negative binomial. By applying some calculus, one can show that the Poisson distribution is a special case of the negative binomial distribution when $\alpha \to \infty$ and $\theta \to 0$, such that the product, $\alpha \theta = {\lambda }$, is kept constant. The parameter ${\text{a}} = \frac{1}{\alpha }$ is associated with the “extra-Poisson” variation or over-dispersion because $var\left( y \right) = {\lambda } + a {{\lambda }}^{2}$, which is quadratic in the mean, that is why the negative binomial model is referred to as the NB2 model. This interpretation justifies a $\left( {{\lambda },{\text{ a}}} \right)$ parameterization of the NB distribution as

$$P_{r} \left( {Y = k;{\lambda },{\text{ a}}} \right) = \left( {\begin{array}{*{20}c} {k + \frac{1}{a} - 1} \\ k \\ \end{array} } \right)\left( {\frac{{{{a\lambda }}}}{{1 + {{a\lambda }}}}} \right)^{k} \left( {\frac{1}{{1 + {{a\lambda }}}}} \right)^{\frac{1}{a}} ,$$

where ${ }E\left[ y \right] = {\lambda }$ and ${\text{var}}\left[ {\text{y}} \right] = {\lambda } + {{a\lambda }}^{2}$, and $a = 0$ results in Poisson distribution. This latest parameterization is useful to specify the NB regression and for testing over-dispersion as $H_{0} :a = 0$^32^.

The likelihood function for Eq. (2) is proportional to

$$L\left( {{\varvec{\beta}}, \alpha } \right) = \mathop \prod \limits_{i = 1}^{n} \frac{{\Gamma \left( {\alpha + k_{i} } \right)}}{{k_{i} ! \Gamma \left( \alpha \right)}}\left( {\frac{{\theta_{i} }}{{1 + \theta_{i} }}} \right)^{{k_{i} }} \left( {\frac{1}{{1 + \theta_{i} }}} \right)^{\alpha }$$

Lawless^32^ notes that for any $c > 0, \Gamma \left( {k + c} \right)/ \Gamma \left( c \right) = c\left( {c + 1} \right)\times \cdots \times \left( {c + k - 1} \right)$ for integer-valued $k \ge 1$, thus, $\frac{{ \Gamma \left( {\alpha + k} \right)}}{ \Gamma \left( \alpha \right)} = \alpha \left( {1 + \alpha } \right)\times \cdots \times \left( {k - 1 + \alpha } \right)$. Hence, $log\left\{ {\frac{{ \Gamma \left( {\alpha + k} \right)}}{ \Gamma \left( \alpha \right)}} \right\} = \mathop \sum \limits_{j = 0}^{{k_{i} - 1}} {\log}\left( {\alpha + j} \right).$ This produces $\log L\left( {{\varvec{\beta}}, \alpha } \right)$ as follows

$$\begin{gathered} = \mathop \sum \limits_{i = 1}^{n} \left( {\mathop \sum \limits_{j = 0}^{{k_{i} - 1}} {\log}\left( {\alpha + j} \right) - \log k_{i} ! + k_{i} \log \theta_{i} - k_{i} \log \left( {1 + \theta_{i} } \right) + \alpha \log 1 - \alpha \log \left( {1 + \theta_{i} } \right)} \right) \hfill \\ \ell \left( {{\varvec{\beta}}, \alpha } \right) = \mathop \sum \limits_{i = 1}^{n} \left( {\mathop \sum \limits_{j = 0}^{{k_{i} - 1}} {\log}\left( {\alpha + j} \right) - \log k_{i} ! + k_{i} \log \theta_{i} - \left( {k_{i} + \alpha } \right)\log \left( {1 + \theta_{i} } \right)} \right) \hfill \\ \end{gathered}$$

Therefore, applying the Poisson theorem with Gamma distribution leads to the negative binomial distribution. Furthermore, detailed discussions of estimating methods and characteristics of the negative binomial model are presented in numerous literature^13,14,25,30–32^.

When repeated counts are measured on the same individual over time, the assumption of independence is no longer reasonable; instead, they are correlated. Subject-specific random effects can be added into the linear predictor to modeling such dependence. Let $y_{ij}$ be the values of a count variable (non-negative integer value) for subject $i$ at time point $j$. The count is assumed to be drawn from a Poisson distribution with errors assumed to have a normal distribution, $\varepsilon_{ij} \sim N\left( {0, \sigma_{\varepsilon }^{2} } \right)$. Then, the Poisson mixed-effects model that specifies the expected number of counts is written as

5 $$\log \left( {\mu_{ij} } \right) = {\varvec{x}}_{ij}^{^{\prime}} {\varvec{\beta}} + {\varvec{z}}_{ij}^{^{\prime}} {\varvec{b}}_{i} + \varepsilon_{ij} ,$$

where ${\varvec{x}}_{ij}$ is the variable of interest, ${\varvec{\beta}}$ is the vector of fixed effects (population-level effects), including an intercept $\beta_{0}$, ${\varvec{b}}_{i}$ is the vector of random effects (subject-level effects) for the sample variables ${\varvec{z}}_{ij}$, and $\varepsilon_{ij}$ is the random errors^22,23^. Given the Poisson process for the count $y_{ij}$, the probability that $y_{ij} = y$, conditionally on the random effects ${\varvec{b}}_{i}$, is given by

$$\begin{gathered} P\left( {y_{ij} = y{|}{\varvec{b}}_{i} ,{\varvec{x}}_{ij} , {\varvec{z}}_{ij} } \right) = \frac{{e^{{ - \mu_{ij} }} \mu_{ij}^{y} }}{y!} = \frac{1}{y!}e^{{ - {\exp}\left( {{\varvec{x}}_{ij}^{{\prime }} {\varvec{\beta}} + {\varvec{z}}_{ij}^{{\prime }} {\varvec{b}}_{i} } \right)}} {\exp}\left( {{\varvec{x}}_{ij}^{{\prime }} {\varvec{\beta}} + {\varvec{z}}_{ij}^{{\prime }} {\varvec{b}}_{i} } \right)^{y} \hfill \\ = \frac{1}{y!}\exp \left[ {\left( {{\varvec{x}}_{ij}^{{\prime }} {\varvec{\beta}} + {\varvec{z}}_{ij}^{{\prime }} {\varvec{b}}_{i} } \right)^{y} - {\exp}\left( {{\varvec{x}}_{ij}^{^{\prime}} {\varvec{\beta}} + {\varvec{z}}_{ij}^{^{\prime}} {\varvec{b}}_{i} } \right)} \right], y = 0, 1, 2, \ldots \hfill \\ \end{gathered}$$

This addition also can be applied to the NBMM that allows over-dispersion by assuming a gamma distribution for the errors; instead of a normal distribution. Suppose that ${\varvec{x}}_{ij}$ and ${\varvec{z}}_{ij}$ are known vectors of covariates associated with count data $y_{ij} , \quad i = 1, \ldots ,n \quad \text{and} \quad j = 1, \ldots , n_{i}$, conditional on a $q-$ dimensional vector of subject-specific random effects, ${\varvec{b}}_{{\varvec{i}}}$, the counts of $y_{ij}$, with the assumption of gamma errors, has a negative binomial distribution, $y_{ij} |{\varvec{b}}_{{\varvec{i}}} \sim NB\left( {\mu_{ij} , \mu_{ij} + \theta \mu_{ij}^{2} } \right)$, with $\mu_{ij} = E\left( {y_{ij} {|}{\varvec{b}}_{{\varvec{i}}} } \right) = {\exp}\left\{ {{\varvec{x}}_{ij}^{^{\prime}} {\varvec{\beta}} + {\varvec{z}}_{ij}^{^{\prime}} {\varvec{b}}_{i} } \right\}$. This indicates that the mean parameters $\mu_{ij}$ of the negative binomial mixed-effects models are also related to the predictor variables ${\varvec{x}}_{ij}$, and the sample variables ${\varvec{z}}_{ij}$ through the logarithm link function: $\log \left( {\mu_{ij} } \right) = {\varvec{x}}_{ij}^{^{\prime}} {\varvec{\beta}} + {\varvec{z}}_{ij}^{^{\prime}} {\varvec{b}}_{i} + \varepsilon_{ij}$, which shows that the model for the conditional mean of the NBMM is similar to that of PMM. However, the conditional variance of $y_{ij}$ for NBMM is $Var\left( {y_{ij} {|}{\varvec{b}}_{{\varvec{i}}} } \right) = \mu_{ij} + \theta \mu_{ij}^{2}$, which is greater than the conditional mean of PMM by $\theta \mu_{ij}^{2}$, specifically, because a gamma distribution is assumed for the exponentiated errors, ${\exp}\left( {\varepsilon_{ij} } \right)$, with a mean of $1$ and variance $\theta$^22,31^. Random effects are used to demonstrate multiple assets of variations and subject-specific effects. As a result, they avoid biased inference on the fixed effects. The random effects are assumed to have a multivariate normal distribution:

6 $${\varvec{b}}_{i} \sim N\left( {0, \Psi } \right)$$

where $\Psi$ is a positive-definite variance–covariance matrix that accounts for the correlation of the random effects^33,34^.

### Ethics approval and consent to participate

Ethical approval for the study was obtained from the Research Ethics Committee of the University of KwaZulu-Natal (E013/04), the University of the Witwatersrand (MM040202), and the University of Cape Town (025/2004). All participants provided written informed consent. All methods were performed following the relevant guidelines and regulations expressed in the Declaration of Helsinki.

## Results

Table 1 shows the summary of CD4 count and its associated selected covariates in the CAPRISA 002 AI Study. The dataset included 235 subjects (7129 observations consists of a minimum of two and a maximum of sixty-one observations per subject). P-values demonstrated in Table 1 are obtained from the Chi-square test. At a 5% level of significance, the univariate cross-tabulation analysis uncovers that the patient’s baseline BMI, baseline VL, number of sexual partners, age, ART initiation, and education level are significantly associated with patient’s CD4 count. Table 1 demonstrates that there is a high prevalence of CD4 count above 500 cells/mm^3^ among patients with normal weight and overweight status, which are 38.32 and 9.36%, respectively (p-value < 0.0001). Out of 7129 observations, patients with an undetectable viral load at baseline indicate no sign of a CD4 count < 500 cells/mm^3^ throughout the study.

**Table 1:** Distribution of CD4 count and associated selected covariates with percent missing.

Covariates	Level	CD4 count N (%)			p-value	% Missing
		< 200	200–500	> 500		
Baseline BMI category	Underweight	2 (0.03)	219 (3.12)	254 (3.62)	< 0.0001	0.0
	Normal weight	114 (1.62)	2305 (32.84)	2690 (38.32)		
	Overweight	18 (0.26)	512 (7.29)	657 (9.36)		
	Obese	0	17 (0.24)	231 (3.29)		
Baseline viral load	Undetected	0	0	16 (0.23)	< 0.0001	0.0
	Low	20 (0.28)	791 (11.27)	1532 (21.83)		
	Medium	45 (0.64)	1209 (17.22)	1497 (21.23)		
	High	69 (0.98)	1053 (15)	787 (11.21)		
Number of sexual partners	No partner	29 (0.41)	565 (8.05)	579 (8.25)	< 0.0001	0.0
	Stable partner	85 (1.21)	2274 (32.4)	3078 (43.85)		
	Many partners	20 (0.28)	214 (3.05)	175 (2.49)		
Age group	< 20	1 (0.01)	130 (1.82)	121 (1.72)	< 0.0001	0.0
	20–29	97 (1.38)	1872 (26.67)	1977 (28.17)		
	30–39	17 (0.24)	813 (11.58)	1255 (17.88)		
	40–49	19 (0.27)	203 (2.89)	369 (5.26)		
	50–59	0	35 (0.5)	91 (1.3)		
	≥ 60	0	0	19 (0.27)		
Educational level	Primary school	3 (0.04)	104 (1.48)	181 (2.58)	0.0129	0.0
	Secondary school	131 (1.87)	2949 (42.01)	3651 (52.02)		
Place of residence	Rural	62 (0.88)	1467 (20.90)	1806 (25.73)	0.7176	0.06
	Urban	72 (1.03)	1586 (22.6)	2026 (28.86)		
ART initiation group	Pre ART	110 (1.57)	2566 (36.56)	2783 (39.65)	< 0.0001	0.0
	Post ART	20 (24)	487 (6.94)	1049 (14.95)		

The response variable (CD cell count) has 110 (1.5%) missing observations.

Moreover, from Table 1, there is a high prevalence of CD4 count above 500 cells/mm^3^ for patients with low viral load at baseline (21.83%). This shows ART suppresses the amount of HIV viably in patient’s body fluids who have an undetectable and low viral load at baseline to the point where standard tests are incapable of detecting any HIV or can only find a little flow. There is also a high prevalence of CD4 count above 500 cells/mm^3^ for patients with a stable sexual partner (43.85%, p-value < 0.0001) compared to patients who have many sexual partners. A high prevalence of CD4 count above 500 cells/mm^3^ is observed among patients of the age group between 20–29 years and 30–39 years, which are 28.17 and 17.88%, respectively (p-value < 0.0001). The prevalence of CD4 count above 500 cells/mm^3^ is also observed among women patients who have higher/secondary school levels of education (52.02%, p-value = 0.0129). However, the place of residence is found not to be associated with patients’ CD4 count (p-value = 0.7176).

**Figure 1:**
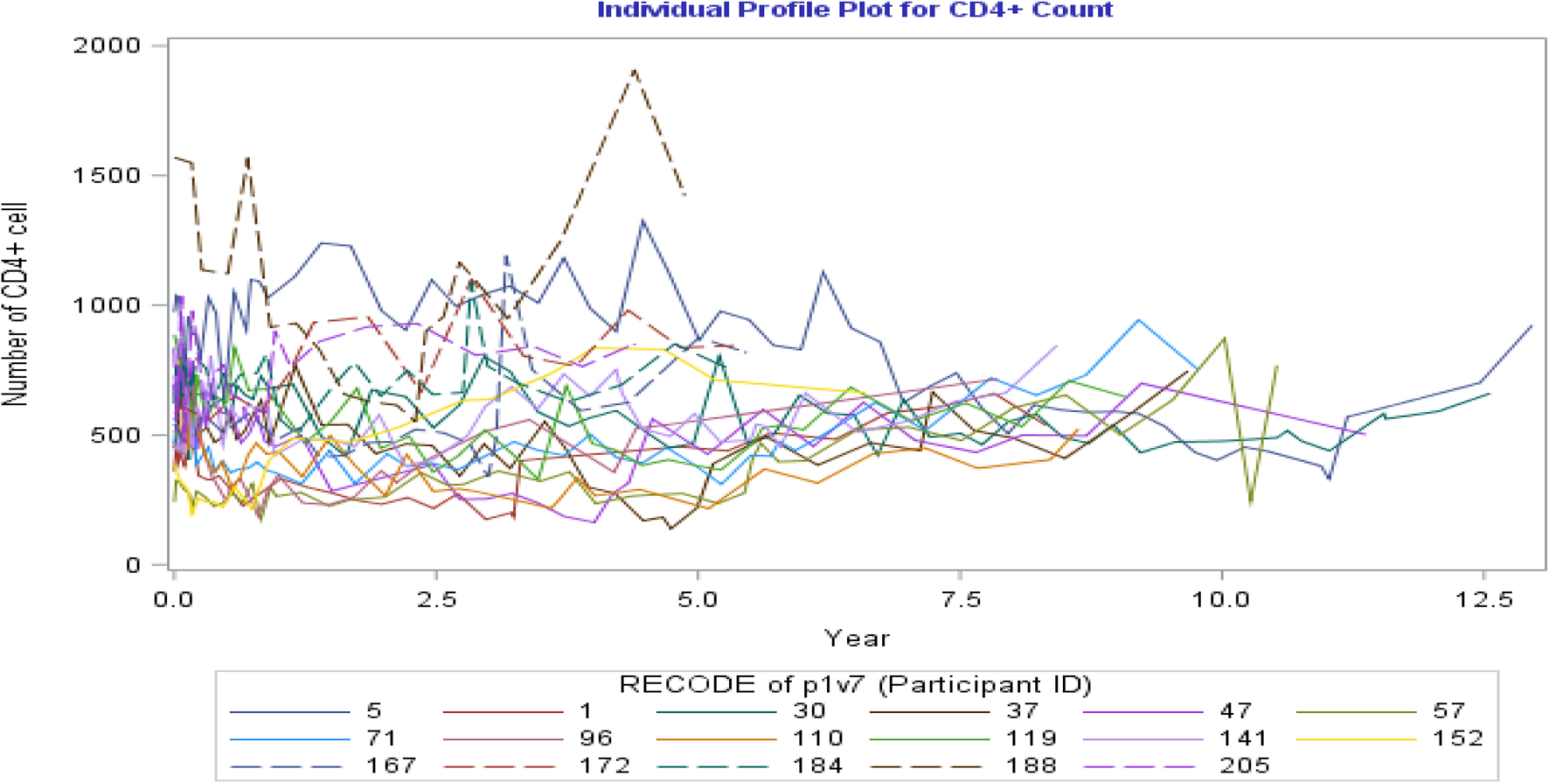
Individual profiles plot of CD4 cell count for 17 randomly selected subjects.

The individual profiles plot for 17 randomly selected HIV-Infected women enrolled in the CAPRISA 002 AI Study is shown in Fig. 1.

Analyzing data shown in Fig. 1, we can observe insights concerning the variability among individual patients at a given point in time, the variance within units over time, and the trends over time. Note that the space between the lines represents between unit variability, and the change in each line (slope) represents within variability. Moreover, as portrayed in Fig. 1, the number of CD4 cells seems to represent a slightly increasing pattern over time; however, the rate of increment is low. Additionally, Fig. 1 shows that there is wide variability in the number of CD4 cells and in the number of repeated measures (number of observations per subjects are not equal).

The results of the Fit statistics in Table 2 are obtainable because of method = Laplace in Proc Glimmix Procedure. These values are relative and valuable when we compare different model choices. The NB model’s Fit statistics are much smaller than the Poisson model (Table 2). For instance, AICC is 87833.48 for NB versus 204893.1 for the Poisson. Also, the Pearson ${\chi }^{2}$/DF of 20.66 for the Poisson model is problematic (Table 3), indicating evidence of over-dispersion in the data. Ideally, this value ought to be generally 1.0 when modeling count data with a Poisson distribution. The ratio of Pearson Chi-Square statistics is dropped from 20.66 to 0.91 under the NB model, which is close to one (Table 3), indicating that over-dispersion has been appropriately modeled and it is no longer an issue under the NB model.

**Table 2:** Comparisons of fit statistics for the two distributions.

Distribution	Fit statistics					
	− 2 log likelihood	AIC	AICC	BIC	CAIC	HQIC
Poisson	204,842.9	204,892.9	204,893.1	204,979.4	205,004.4	204,927.8
NB	87,781.28	87,833.28	87,833.48	87,923.23	87,949.23	87,869.54

**Table 3:** Measure of over-dispersion between Poisson and negative binomial distribution.

Fit Statistics for Conditional Distribution	Poisson	NB
− 2 log L(CD4 counts/r. effects)	199,670.3	85,320.39
Pearson $\chi^{2}$	145,017.0	6396.89
Pearson $\chi^{2}$/DF	20.66	**0.91**

**Figure 2:**
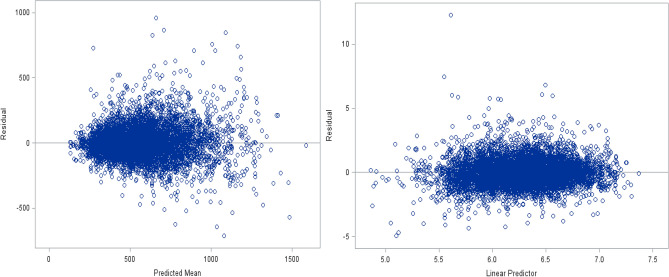
Data-scale raw residuals and Model-scale studentized residuals versus predicted values.

In addition to the conditional fit statistics, any other diagnostic that may allow us to see over-dispersion in the Poisson model is a graphical representation (Fig. 2). We can get residual plots through Proc Glimmix using the Plot option. Here, we only focus on looking at residual versus predicted plots. Figure 2 (left panel) shows the visual prove of over-dispersion. As the Predicted Mean ($\hat{\mu }$) increases, the associated residuals become more broadly dispersed. The variance ought to increase as a function of the mean, but not as quickly as we see in this plot (Fig. 2). Also, Fig. 2 (right panel) shows prove of over-dispersion. The variance adjusted residuals are more variable around the lower point of the estimated Linear Predictor $(\hat{\eta }$). On the model scale (Fig. 2 (right panel)), we should not see the variance adjusted residuals variable across different points of $\hat{\eta }$ as we see in this plot^16,35^. In other words, Fig. 2 (right panel) demonstrates that the empirical distribution of the residuals is not reasonably symmetric, and in general, it is not very informative.

The improvement in the Pearson ${\chi }^{2}$/DF and Fit statistics indicate that it is best to model data from this experiment with the NB distribution. Utilizing the proper distribution gives unbiased test statistics and SE estimates (Table 4).

**Table 4:** Comparison of random effect models.

Random effect models	Information criteria					
	− 2log ${ }\ell$	AIC	AICC	BIC	CAIC	HQIC
Model 1	87,781.28	87,833.28	87,833.48	87,923.23	87,949.23	87,869.54
Model 2	88,603.50	88,649.50	88,649.66	88,729.07	88,752.07	88,681.58
Model 3	88,591.64	88,637.64	88,637.80	88,717.21	88,740.21	88,669.72
Model 4	89,156.39	89,202.39	89,202.55	89,281.96	89,304.96	89,234.47
Model 5	89,837.18	89,879.18	89,879.31	89,951.83	89,972.83	89,908.47
Model 6	92,302.08	92,344.08	92,344.21	92,416.73	92,437.73	92,373.37
Model 7	91,190.61	91,232.61	91,232.74	91,305.26	91,326.26	91,261.90

In addition, the subsequent random effect models were taken into consideration for testing NBMMs:

Model 1: *Intercept, Time,*$\sqrt {Time}$.

Model 2: *Intercept, Time.*

Model 3: *Intercept,* $$\sqrt {Time}$$.

Model 4: *Time,* $$\sqrt {Time}$$.

Model 5: *Intercept only.*

Model 6: *Time only.*

Model 7: $$\sqrt {Time}$$*only.*

We conclude that Model 1 is a preferable model among models listed above since it has the smallest information criteria. Moreover, a comparison of the covariance structure using the fitted model (Supplementary Table S1) and a comparison of fixed-effects results across different covariance structures using Model 1 (Supplementary Table S2) are made. The estimated unstructured covariance matrix ($\hat{D}$) for the GLMMs model that uses NB distribution is

$$\hat{D} = \left[ {\begin{array}{*{20}c} {0.1131} & {\begin{array}{*{20}c} {0.000739} & { - 0.01754} \\ \end{array} } \\ {\begin{array}{*{20}c} {0.000739} \\ { - 0.01754} \\ \end{array} } & {\begin{array}{*{20}c} {\begin{array}{*{20}c} {0.000155} \\ { - 0.00137} \\ \end{array} } & {\begin{array}{*{20}c} { - 0.00137} \\ {0.01556} \\ \end{array} } \\ \end{array} } \\ \end{array} } \right]$$

The estimated scale parameter is 0.04205, which can be found in the “Covariance Parameter Estimates” output of the SAS PROC GLIMMIX (Laplace) procedure (see Supplementary Table S3). Therefore, the estimated conditional variance of the count is $\hat{\mu }_{i} + 0.04205\hat{\mu }_{i}^{2}$, where $\hat{\mu }_{i}$ is the conditional mean on the counting scale. “The Scale parameter measures the magnitude of over-dispersion and is practically equivalent to the mean square error in conventional theory analysis of variance”^15^.

Table 5 shows the overall effect of the selected factors within the fitted models. The results indicate that the effects of Time, Baseline BMI, HAART initiation group, baseline viral load, and the number of sexual partners on the patient’s CD4 count were found to be highly significant in both fitted models. However, the overall F-values of the NB model were smaller than for the Poisson model. This can be supporting prove that over-dispersion can lead to inflated and biased F-values if we do not use the proper model in our analysis.

**Table 5:** Type III Analysis of fixed effects for Poisson and NB distribution.

Effect	Num DF	Den DF	NB		Poisson	
			F value	Pr > F	F value	Pr > F
Time in month	1	235	62.53	< 0.0001	14.80	0.0002
Sqrt_Time	1	234	86.36	< 0.0001	48.41	< 0.0001
Baseline BMI category	3	6307	6.26	0.0003	6.31	0.0003
ART initiation	1	6307	345.45	< 0.0001	5890.28	< 0.0001
Baseline VL	3	6307	7.48	< 0.0001	12.79	< 0.0001
No. of sexual partners	2	6307	1.64	0.1935	1.85	0.1578
Age group	5	6307	1.46	0.1987	27.34	< 0.0001
Education level	1	6307	0.25	0.6196	0.15	0.6990
Place of residence	1	6307	0.01	0.9246	0.11	0.7406

Table 6 shows the log of the expected CD4 count as a function of the selected predictor variables using a negative binomial mixed-effect model. The results indicate that time (month) significantly affects the CD4 count of a patient. We interpret the coefficient of the month as an average within-subject change in the logs of expected CD4 count for patients would be expected to increase by 0.0078 units (p-value < 0.0001; 95% CI 0.005875, 0.009774), while holding other factors in the model constant. The square root of time shows a significant adverse effect in the logs of expected CD4 counts of a patient (Table 6). Compared to pre HAART initiation, the difference in the logs of CD4 counts of a patient who had been initiated on HAART would be expected to increase by 0.2301 units (p-value < 0.0001; 95% CI 0.2058, 0.2543), holding other factors constant in the model. It can be observed that the difference in the logs of expected CD4 counts is expected to be 0.4815 units (p-value < 0.0001; 95% CI 0.2633, 0.6996) higher for patients with higher BMI (Obese) at baseline compared to patients with normal weight status holding other factors constant in the model. Those patients who had high and medium viral load at baseline, the difference in the logs of their expected CD4 counts were decreased by 0.2393 (p-value < 0.0001; 95% CI − 0.3404, − 0.1382) and 0.1258 (p-value = 0.0061; 95% CI − 0.2157, − 0.03585), respectively, compared to patients who had low viral load at baseline while holding other factors in the model constant.

**Table 6:** Parameter estimates using Poisson and NB mixed-effects model.

Covariates	Negative binomial mixed-effects model				Poisson mixed-effects model		
	Estimate	SE	Pr >|t|	95% CI for NB estimate	Estimate	SE	Pr >|t|
Intercept	6.4697	0.04982	< 0.0001	(6.3715, 6.5679)	6.4625	0.04264	< 0.0001
Time in month	0.007824	0.000989	< 0.0001	(0.005875, 0.009774)	0.006564	0.001706	0.0002
Sqrt_Time	− 0.08649	0.009307	< 0.0001	(− 0.1048, − 0.06815)	− 0.06839	0.009830	< 0.0001
ART initiation (post)	0.2301	0.01238	< 0.0001	(0.2058, 0.2543)	0.1947	0.002537	< 0.0001
**Baseline BMI category (ref. = normal weight)**							
Obese	0.4815	0.1113	< 0.0001	(0.2633, 0.6996)	0.4985	0.1147	< 0.0001
Overweight	0.02561	0.04975	0.6067	(− 0.07191, 0.1231)	0.03131	0.05148	0.5431
Underweight	0.005901	0.07927	0.9407	(− 0.1495, 0.1613)	0.01691	0.08264	0.8379
**Baseline HIV viral load category (ref. = low VL)**							
High VL	− 0.2393	0.05157	< 0.0001	(− 0.3404, − 0.1382)	− 0.3074	0.05065	< 0.0001
Medium VL	− 0.1258	0.04587	0.0061	(− 0.2157, − 0.03585)	− 0.1121	0.04686	0.0168
Undetectable	0.1377	0.2901	0.6351	(− 0.4310, 0.7064)	0.1199	0.2978	0.6872
**Number of sexual partners (ref. = stable partner)**							
Many partners	− 0.1560	0.09394	0.0967	(− 0.3402, 0.02811)	− 0.1674	0.09908	0.0911
No partner	− 0.04821	0.04993	0.3343	(− 0.1461, 0.04967)	− 0.05913	0.05164	0.2522
**Age group in years (ref. = < 20)**							
20–29	0.01166	0.03104	0.7072	(− 0.04919, 0.07251)	− 0.00791	0.007830	0.3125
30–39	0.02852	0.03432	0.4060	(− 0.03876, 0.09580)	− 0.01239	0.008474	0.1438
40–49	− 0.00719	0.04545	0.8743	(− 0.09629, 0.08191)	− 0.03422	0.01112	0.0021
50–59	− 0.05694	0.06662	0.3927	(− 0.1875, 0.07365)	− 0.1399	0.01549	< 0.0001
≥ 60	0.2082	0.1532	0.1741	(− 0.09205, 0.5084)	− 0.3107	0.03519	< 0.0001
**Education attainment (ref. = secondary or high school)**							
Primary school	− 0.04509	0.09084	0.6196	(− 0.2232, 0.1330)	− 0.03582	0.09263	0.6990
**Residence of participant (ref. = urban)**							
Rural	− 0.00373	0.03947	0.9246	(− 0.08112, 0.07365)	0.01337	0.04038	0.7406

Furthermore, the SEs for the Poisson mixed-effects model were more likely to be underestimated and/or biased compared to those from a negative binomial mixed-effects model since the model is fitted by ignoring over-dispersion of the data (Table 6).

The prediction profile equation for the average number of CD4 cell following Table 6 results obtained by NB mixed-effects model is given as:

$$\begin{aligned} log\left( {\hat{\mu }_{i} } \right) & = 6.4697 + 0.007824 \times time - 0.08649 \times \sqrt {time} + 0.2301 \\ & \quad \times post HAART treatment + 0.4815 \times obese - 0.2393 \\ & \quad \times high VL - 0.1258 \times medium VL. \\ \end{aligned}$$

Taking antilog values on both sides of the above-predicted equation yields the expected number of counts, given by

$$\hat{\mu }_{i} { } = \exp \left( {6.4697 + 0.007824 \times time - 0.08649 \times \sqrt {time} + 0.2301 \times post HAART treatment + 0.4815 \times obese - 0.2393 \times high VL - 0.1258 \times medium VL} \right).$$

**Figure 3:**
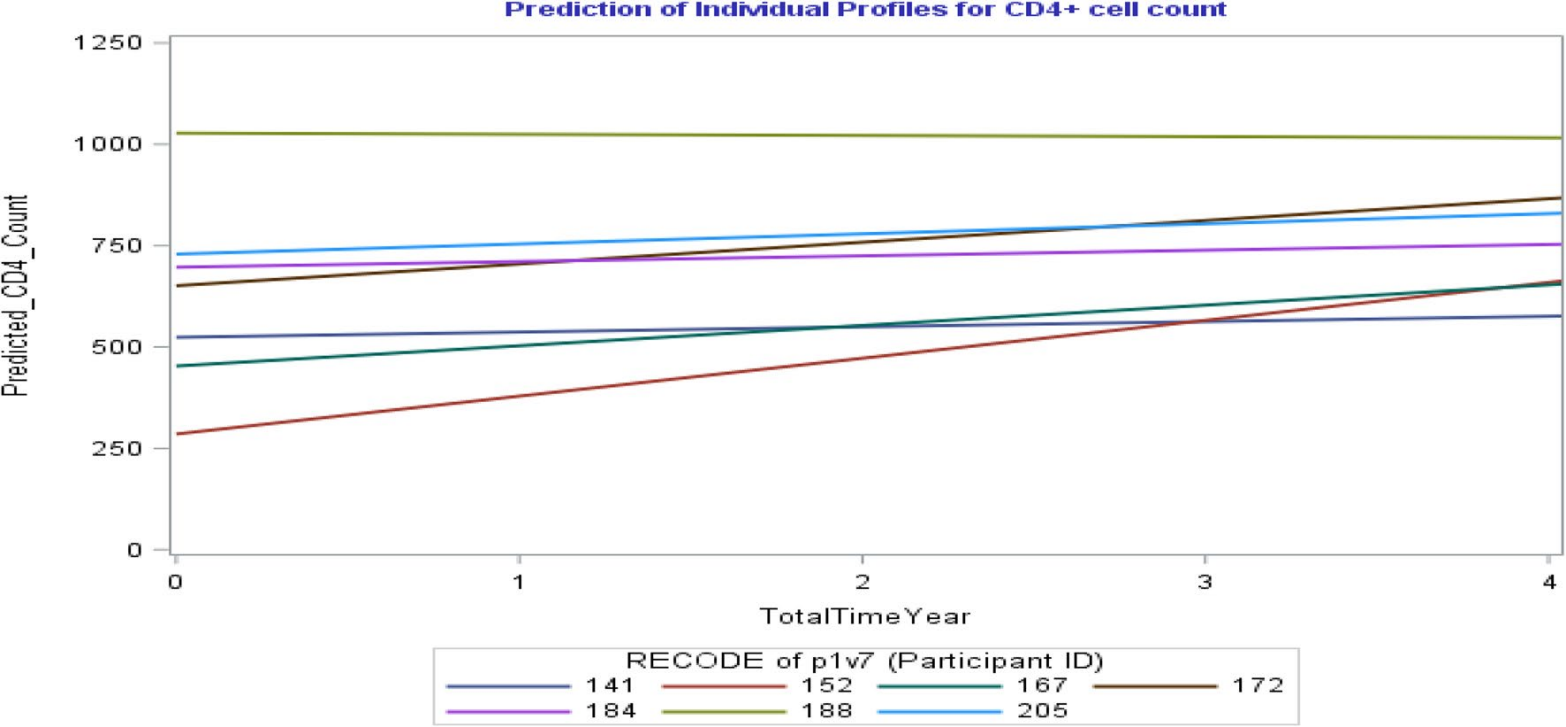
Prediction of 7 randomly selected individual profiles plot of CD4 count for 4 years.

The prediction of individual profiles, Fig. 3, presents the estimated trajectories for the average number of CD4 cell under the estimates acquired by the negative binomial mixed-effect model with UN covariance structure consolidated with the model where the intercept and slope were considered as random effects (see Table 4 and Supplementary Table S1) for seven patients with particular profiles for four years. For instance, from CAPRISA 002 AI Study, patient ID = 141, 22 years old female, with around 500 cells/mm^3^ CD4 cell count at baseline, low VL at baseline, had normal weight status at baseline, and have no sexual partner at the time of enrollment.

The second patient ID = 152, 34 years old female, with obese weight status at baseline, having stable sexual partner, high VL at baseline, and CD4 count at baseline below 500 cells/mm^3^. As a third example, we looked at patient ID = 172 who had undetected VL at baseline, with CD4 count at baseline above 500 cells/mm^3^, 29 years old female, with obese weight status at baseline and have a stable sexual partner. As a fourth example, we can also look at patient ID = 188, who had a high number of CD4 cells at baseline (1070 cells/mm^3^) with low VL at baseline, 42 years old, had obese weight status at baseline, and have a stable sexual partner. As we would anticipate, all seven individuals appeared to have an increased average number of CD4 cells over time, in line with their predicted individual profiles (Fig. 3). However, the increasing level or degree is different among individuals. This is due to factors related to this study and numerous other characteristics of these individuals, mainly (according to our research) for their VL at baseline, baseline BMI and the treatment (either the patient had effective HAART initiation after HIV exposure or not).

Moreover, for this study to yield meaningful results, we checked the missing values in the dataset using the Little’s MCAR test. The regular Little’s MCAR test gives us a χ^2^ distance of 4515.686 with a degree of freedom 106 and p-value 0.000 (Little’s MCAR test: Chi-Square = 4515.686, DF = 106, sig. = 0.000). The analysis gives evidence that the missing data in the study variables of interest are not MCAR under significance level 0.000. Therefore, we used Multiple Imputation (MI) techniques to get a valid analysis for parameter estimates from the complete data set by fitting the chosen model. The MI procedure’s main concept is to replace each missing value with a set of *m* possible values. Generally, the imputation of dependent and independent variables is basic for getting unbiased estimates of the regression coefficients^36^. Following Rubin’s (1987) terminology, the MI procedure includes three distinct phases: each missing value is imputed *m* times to generate *m* complete data sets, analyze each *m* complete data sets separately by using standard procedure and then combine the results to generate valid statistical inference about the model parameters from the *m* data set analysis using Rubin’s combine rule^37^. SAS Proc MI can be used to create *N* number of imputation; after that, Proc MIAnalyze is used to pool the parameter estimates. A detailed discussion of missing data analysis and how missing data handled by statistical software can be found in numerous literature^37–44^.

Table 7 shows a combined result for each parameter. The table also shows a 95% confidence interval, the minimum and maximum regression coefficients from the imputed data set, and the associated p-value. We can compare the results given in Table 7 with the results of applying the negative binomial mixed-effect model to the CAPRISA 002 AI data using incomplete cases (Table 6). Comparing the two different sets of results, we do not see that many exciting differences. In both cases, covariates that were found to be significantly affecting the patient’s CD4 count are similar, and their respective parameter estimates are more close to each other.

**Table 7:** Combined results of a negative binomial mixed-effects model analysis using MI Procedure to deal with the missing values.

Parameter	Parameter estimates (10 imputations)					
	Estimate	SE	Pr >|t|	95% confidence limits	Minimum	Maximum
Intercept	6.459413	0.049830	< 0.0001	(6.36175, 6.55708)	6.458658	6.460775
Time in month	0.007475	0.000975	< 0.0001	(0.00556, 0.00939)	0.007450	0.007508
Sqrt_Time	− 0.083647	0.009266	< 0.0001	(− 0.10181, − 0.06549)	− 0.083982	− 0.083434
ART initiation (Post)	0.224037	0.012594	< 0.0001	(0.19935, 0.24872)	0.223216	0.225014
**Baseline BMI category (ref. = normal weight)**						
Obese	0.474714	0.109902	< 0.0001	(0.25931, 0.69012)	0.473892	0.475630
Overweight	0.024208	0.048971	0.6211	(− 0.07177, 0.12019)	0.023820	0.024529
Underweight	0.002070	0.078101	0.9789	(− 0.15101, 0.15515)	0.001321	0.003137
**Baseline HIV viral load category (ref. = Low VL)**						
High VL	− 0.239102	0.051294	< 0.0001	(− 0.33964, − 0.13857)	− 0.239735	− 0.238839
Medium VL	− 0.122078	0.045390	0.0072	(− 0.21104, − 0.03311)	− 0.122251	− 0.121642
Undetectable	0.142848	0.286259	0.6178	(− 0.41821, 0.70391)	0.142510	0.143351
**Number of sexual partners (ref. = stable partner)**						
Many partners	− 0.153632	0.092090	0.0953	(− 0.33412, 0.02686)	− 0.154667	− 0.152911
No partner	− 0.046962	0.049227	0.3401	(− 0.14344, 0.04952)	− 0.047267	− 0.046691
**Age group in years (ref. = < 20)**						
20–29	0.013477	0.031659	0.6703	(− 0.04857, 0.07553)	0.012306	0.014325
30–39	0.033725	0.034974	0.3349	(− 0.03482, 0.10227)	0.032678	0.034744
40–49	− 0.005842	0.046177	0.8993	(− 0.09635, 0.08466)	− 0.007790	− 0.004745
50–59	− 0.052070	0.067501	0.4405	(− 0.18437, 0.08023)	− 0.054207	− 0.051024
≥ 60	0.206708	0.156046	0.1853	(− 0.09914, 0.51255)	0.205360	0.207553
**Education attainment (ref. = secondary or high school)**						
Primary school	− 0.046292	0.089605	0.6054	(− 0.22191, 0.12933)	− 0.046602	− 0.046009
**Residence of participant (ref. = urban)**						
Rural	− 0.001916	0.038813	0.9606	(− 0.07799, 0.07416)	− 0.002146	− 0.001596

In general terms, a comparison of the results from data with missing value case analysis (Table 6) and multiple imputation analysis (Table 7) shows little difference between parameter estimates, SEs, and confidence intervals. In this case, the small difference in results and associated inferences is likely due to relatively low amounts of missing data in the analysis variables (Table 1). However, it will not always be true that results from incomplete or complete case analysis and a multiple imputation treatment of the data will lead to similar results and inferences^38^. Finally, missing data is especially common in longitudinal data sets. Missingness can arise due to respondent attrition, survey structure, file-matching issues, and refusal to answer sensitive questions such as certain health conditions, illegal behaviors, or income^38^. Missing data can also arise due to death. A loss to follow-up due to death is qualitatively different from dropout due to other responses and, ordinarily, needs to be dealt with quite differently in the analysis of longitudinal data^9^. Missing data is generally classified as Missing Completely at Random (MCAR), Missing at Random (MAR), or Not Missing at Random (NMAR)^37,39,41,44–46^.

## Discussion and conclusion

GLMs extend the standard concept of linear models to outcome variables whose distribution is from a member of the exponential family. “GLM consists of three components: a *stochastic* component that characterizes the likelihood distribution of the response variable; a *linear predictor* that is a *systematic* component portraying the linear model characterized by the explanatory variables; and a *link function* that connect the mean of the response variable to a linear combination of the explanatory variables. Link functions that are commonly used for distributions are discussed in numerous literature”^12,16,24,28,35,47–51^. Parameters in GLM are estimated based on maximum likelihood principles. Different ways of transformations of the response variable make the transformed data to fulfill the linear model’s assumptions, such as approximately normally distributed and having stable variances. In a more common term, a transformation is a replacement that changes the shape of distribution or relationship. However, transformation is often challenging for regression settings in which it additionally influences the practical relationship between the covariates and the outcome variable. In some cases, it is not perceived that the utilization of transformations changes the model^52^.

Transformations are elaborative when a selected choice is not predetermined through different considerations; that is, the selection of transformation is subjective^53^. “GLMs avoid these problems since the data are no longer transformed; instead, a function of the means is modeled as a linear combination of the covariates”^24,48^. Sometimes, for example, for large values of the estimated coefficient, the use of a transformation is effective than using GLMs and Wald type statistics for inference^48,49^. “In general, however, transformations rarely compete well with GLMs for adequately powered studies”^48^. Therefore, we analyzed the non-normal untransformed form of the CD4 cell count of a patient enrolled in the CAPRISA 002 AI Study in the context of GLMMs (Table 6).

Longitudinal studies, also called mixed-effects models, are used to study changes in the response variable over a relevant interval of time or space and the effects of different factors on these changes. The two fundamental issues in longitudinal studies are constructing an appropriate model for the mean and choosing a reasonable but parsimonious model for the covariance structure of longitudinal data^22^. For these reasons, we have fitted an NBMM consolidated with the UN covariance structure since there was enough evidence of over-dispersion in the data. The chosen covariance structure gives the smallest information criteria (Supplementary Table S1). The comparisons between Poisson and negative binomial mixed-effects models were outlined in Table 6.

Moreover, comparisons of the covariance structure illustrated in Supplementary Table S1. GLMMs combine the GLMs with the LMMs. “As an extension of GLMs, they consolidate random effects into the linear predictor. As a mixed model, they contain at least one fixed effect and at least one random effect”^54^. Parameter estimation in GLMMs is also based on maximum likelihood principles; inferences for the parameters are readily obtained from classical maximum likelihood theory^22,54^. “The two fundamental computational methods to attain solutions to the likelihood equations are a *pseudo-likelihood*, and integral approximation of the log-likelihood using either the Laplace or Gauss-Hermite quadrature strategies”^16,40,55^. Since *pseudo-likelihood* generates biased covariance parameter estimates when the number of observations per subject is small, it is especially inclined to biased estimates when the power is small and uses a *pseudo-likelihood* rather than a true likelihood, likelihood ratio, and fit statistics such as AICC and BIC have no clear meaning. However, the integral approximation uses the actual likelihood and grant us the appropriate likelihood ratio tests or information criteria, permitting competing models to be compared using these test statistics. Of these two, the Laplace method is best since quadrature is ordinarily computationally restrictive for regularly repeated measures. Moreover, the Laplace procedure is less computationally intensive than the quadrature procedure and is considerably more flexible in terms of the models with which it can be used. Detailed discussions of parameter estimation in GLMMs can be found in numerous literature^16,22,28,47,48,51^. The fit statistics in Table 3 were obtained by using the Laplace method. If this method had not been specified on the SAS Proc Glimmix procedure, the default *pseudo-likelihood* method would have been used to fit the model. Because *pseudo-likelihood* is based on Tylor series approximation to the conditional likelihood and not expressly on the conditional likelihood itself, a goodness of fit statistic which includes the Pearson ${\chi }^{2}$ that is particularly appropriate to the conditional distribution cannot be computed. Rather, the *pseudo-likelihood* approaches calculate a Generalized ${\chi }^{2}$ statistic that measures the combined fit of the conditional distribution of the counts and the random effects. Since it is not particular to solely the conditional distribution, it does not offer a clear cut diagnostic to evaluate the fit of the Poisson distribution to the counts^40^.

The Pearson ${\chi }^{2}$/DF gives the goodness of fit statistic to evaluate over-dispersion within the Poisson model. Since the variance and mean of the Poisson are equal, the scale parameter (α) is 1. If the Poisson assumption is fulfilled, the Pearson ${\chi }^{2}$/DF ought to be close to 1. Its estimated value of 20.66 (Table 3) indicated solid prove of over-dispersion under the Poisson model. “Over-dispersion would mean more variability shown by the data than would be assumed under a given statistical model”^20^. Over-dispersion could be an issue that should not be disregarded in the statistical inferences. The essential and most critical outcome of over-dispersion is its effect on SEs and test statistics. This was demonstrated in Table 5, uncorrected analysis of over-dispersed data (Poisson model) consequences underestimated SEs, leading to biased estimates and inflated test statistics. “It is basic to check for over-dispersion when fitting a GLM or a GLMM to guarantee that inferences derived from the fitted model are precise”^20^. Over-dispersion is an implication that the fitted model is incorrect, and adjustments are required. “The two most commonly used approaches in GLMMs, to avoid unwanted outcomes outlined above, are: adjusting the SEs and test statistics by incorporating an adjustment for over-dispersion in the model or assume a different probability distribution for the counts that more reasonably approximate the method by which over-dispersion emerge”^48^. Because the second strategy of assuming a different distribution is a reasonable and suggested methodology, it was illustrated in Table 5 in which the negative binomial distribution substitutes the Poisson distribution as the conditional distribution of the outcome. The NB distribution is the foremost candidate as an alternative to the Poisson^13,14^. The Pearson ${\chi }^{2}$/DF value of 0.91 (Table 3) shows that the negative binomial gives a much-improved fit of the data compared to the Poisson model. This is one of a reasonable GLMMs approach for managing with over-dispersion.

Supplementary Table S2 outlined that the fixed effects are significantly influenced by the covariance structure. Furthermore, the covariance structure also impacted the random effects estimate: the time effects and their SEs. The SEs tend to be affected more than the estimates. The selection of covariance structures subjects for non-normally distributed data, just as it does for normally distributed data. The fit statistics related to *pseudo-likelihood* estimation are not comparable among models. Consequently, the fit statistics cannot be used to select between competing for covariance structures. Therefore, the choice of covariance structure is not as straightforward for non-normal longitudinal response data as it is under normality assumption^15,52,55–58^. However, for the GLMM approach, the situation is better. As we discussed previously, since the GLMM characterizes an exact probability process under the Laplace method, fit statistics such as AICC and BIC can be obtained^57^. Thus, for GLMMs, covariance structures selection can continue much as it does for normally distributed data as long as either Laplace (preferable) or quadrature techniques are used. Moreover, while we have incorporated a parametric spatial covariance structure for the fitted negative binomial mixed-effects model, other procedures to account for spatial variation are of interest. Our study methodology, in theory, can be extended to deal with this issue using a GLMM for spatial data^29^. Therefore, we leave this and other attainable extensions for future studies.

Along this line, it would be fascinating to extend this study to the quantile mixed-effects model. Most longitudinal modeling techniques are primarily based on mean regression to focus only on the average effect of covariate and the mean trajectory of the longitudinal outcome, which is constant throughout the population. But, such average effects are not always of interest in lots of study areas and sometimes quite heterogeneous. Thus, quantile mixed-effects model has the capacity, at both the population and individual level, to discover heterogeneous covariates effects, and describe variations in longitudinal studies at different quantiles of the response variable, and hence leads to more efficient estimates, especially when the errors are over-dispersed^59,60^.

## Supplementary information

Supplementary file 1

## Data Availability

The datasets used for this study can be obtained by requesting the corresponding author on reasonable request.

## Abbreviations

AI: Acute Infection
AIDS: Acquired immune deficiency syndrome
ART: Antiretroviral therapy
ARV: Antiretroviral (drug)
CAPRISA: Centre of the AIDS Programme of Research in South Africa
CD4: Cluster of difference 4 cell (T-lymphocyte cell)
GLM: Generalized linear model
GLMM: Generalized linear mixed model
HAART: Highly active antiretroviral therapy
HIV: Human immunodeficiency virus
MI: Multiple imputations
NBMM: Negative binomial mixed-effects model;
PMM: Poisson mixed-effects model
SE: Standard error
STD: Sexually transmitted disease
VL: Viral load refers to the number of HIV copies in a milliliter of blood (copies/ml)
