# Supplementary material for: Negative binomial mixed models for analyzing longitudinal CD4 count data
Source: Sci Rep. 2020 Oct 7;10:16742. doi: 10.1038/s41598-020-73883-7 (PMC7541535; doi:10.1038/s41598-020-73883-7)
Supplement: Supplementary file 1 [file 41598_2020_73883_MOESM1_ESM.pdf]

Negative Binomial Mixed Models for Analyzing Longitudinal CD4 Count Data

Ashenafi A Yirga[1*], Sileshi F Melesse[1], Henry G Mwambi[1]and Dawit G Ayele[2]

1. School of Mathematics, Statistics, and Computer Science, University of KwaZulu-Natal, Pietermaritzburg, Private Bag X01, Scottsville, 3209, South Africa.
2. Institute of Human Virology, University of Maryland, School of Medicine, USA.

*Corresponding Author: Ashenafi Argaw Yirga, Ph.D. Candidate
University of KwaZulu-Natal
Pietermaritzburg, South Africa
Email: ashu3argaw@gmail.com, or 216065934@stu.ukzn.ac.za

Supplementary Table 1 Comparison of covariance structure using the fitted model (Model 1)

**Covariance Structure**	**Information Criteria**					
	-2log ℓ	AIC	AICC	BIC	CAIC	HQIC
AR(1)	89116.94	89116.94	89161.09	89237.05	89259.05	89191.63
CS	89135.76	89179.76	89179.91	89255.87	89277.87	89210.45
Toep	89113.46	89159.46	89159.62	89239.03	89262.03	89191.54
**UN**	**87781.28**	**87833.28**	**87833.48**	**87923.23**	**87949.23**	**87869.54**
VC	88069.85	88115.85	88116.00	88195.42	88218.42	88147.93
ARH(1)	87968.69	88016.69	88016.86	88099.72	88123.72	88050.17
CSH	87893.60	87941.60	87941.78	88024.63	88048.63	87975.08
ToepH	87888.63	87938.63	87938.81	88025.12	88050.12	87973.50

Supplementary Table 2 Comparison of fixed effects results across different covariance structure using Model 1

	**UN**		**AR(1)**		**CS**		**Toep**	
**Covariates**	Estimate	SE	Estimate	SE	Estimate	SE	Estimate	SE
**Intercept**	6.4697	0.04982	6.4724	0.03423	6.4861	0.03410	6.4799	0.03439
**Time in month**	0.007824	0.000989	0.008516	0.01060	0.01439	0.01051	0.008272	0.01082
**Sqrt_Time**	-0.08649	0.009307	-0.08950	0.01180	-0.08434	0.01170	-0.08886	0.01201
**ART Initiation (Post)**	0.2301	0.01238	0.2284	0.01263	0.2363	0.01265	0.2277	0.01264
**Baseline BMI category (ref.=Normal weight)**								
**Obese**	0.4815	0.1113	0.6076	0.07836	0.5097	0.07765	0.6350	0.07813
**Overweight**	0.02561	0.04975	0.02687	0.03466	0.02072	0.03441	0.02970	0.03448
**Underweight**	0.005901	0.07927	0.09673	0.05503	0.03837	0.05470	0.09359	0.05481
**Baseline HIV viral load category (ref.= Low VL)**								
**High VL**	-0.2393	0.05157	-0.3307	0.03345	-0.3234	0.03321	-0.3377	0.03330
**Medium VL**	-0.1258	0.04587	-0.1527	0.03130	-0.1254	0.03112	-0.1567	0.03116
**Undetectable**	0.1377	0.2901	-0.04788	0.2242	0.1338	0.2256	-0.01985	0.2218
**Number of sexual partners (ref.= Stable partner)**								
**Many partners**	-0.1560	0.09394	-0.05213	0.06388	-0.1506	0.06352	-0.04274	0.06393
**No partner**	-0.04821	0.04993	-0.03423	0.03459	-0.05490	0.03434	-0.03322	0.03438
**Age group in years(ref.= < 20)**								
**20-29**	0.01166	0.03104	0.02553	0.02516	0.006652	0.02519	0.02065	0.02543
**30-39**	0.02852	0.03432	0.04911	0.02849	0.03351	0.02850	0.04303	0.02871
**40-49**	-0.00719	0.04545	0.007849	0.04070	0.01926	0.04068	-0.00114	0.04084
**50-59**	-0.05694	0.06662	-0.06551	0.06134	-0.03957	0.06135	-0.06503	0.06143
**≥ 60**	0.2082	0.1532	-0.2185	0.1606	0.2020	0.1601	-0.1844	0.1612
**Education attainment (ref.= Secondary or high school)**								
**Primary school**	-0.04509	0.09084	0.1126	0.06341	-0.00666	0.06299	0.09430	0.06306
**Residence of participant (ref.= Urban)**								
**Rural**	-0.00373	0.03947	0.003881	0.02707	0.01729	0.02689	0.003076	0.02694

Supplementary Table 3 Unstructured covariance Parameter Estimates

Cov Parm	Subject	Estimate
**UN(1,1)**	PID	0.1131
**UN(2,1)**	PID	0.000739
**UN(2,2)**	PID	0.000155
**UN(3,1)**	PID	-0.01754
**UN(3,2)**	PID	-0.00137
**UN(3,3)**	PID	0.01556
**Scale**		0.04205
